# Excite, or Take Flight? Exploring the Relationship between Difficulties with Emotion Regulation, Outcome Expectancies, and Problem Gambling

**DOI:** 10.1007/s10899-024-10340-4

**Published:** 2024-07-25

**Authors:** Annabelle Lee, Mal Flack, Kim M. Caudwell

**Affiliations:** 1 Faculty of Health, Ellengowan Drive, Casuarina, NT 0810 Australia; 2 Researchers in Behavioural Addictions, Alcohol and Drugs, Charles Darwin University, Ellengowan Drive, Casuarina, NT 0810 Australia

**Keywords:** Problem gambling, Emotional dysregulation, Difficulties with emotion regulation, Outcome expectancies, Escape, Excitement

## Abstract

Emotional dysregulation is a transdiagnostic process associated with a range of addictive behaviours including problem gambling, with emerging research indicating that emotionally oriented reasons for gambling (i.e., excitement, escape) are associated with problem gambling. However, the relationships between difficulties with emotion regulation, reasons for gambling, and problem gambling, are unclear. The current study tested whether the association between difficulties with emotion regulation and problem gambling could be explained by escape and excitement gambling outcome expectancies. A total of 187 regular gamblers recruited via social media (50.3% male, 48.7% female) completed measures of difficulties with emotion regulation, gambling outcome expectancies, and problem gambling severity (*M*_*age*_ = 41.07, *SD* = 15.8). Analyses revealed that escape outcome expectancies partially mediated the relationship between difficulties with emotion regulation and problem gambling severity. However, the mediating effect of excitement on this relationship was not significant. The findings suggest that individuals with greater emotional regulation difficulties may engage in problem gambling to help manage aversive emotional states. The study’s findings illustrate the importance of considering emotional dysregulation and outcome expectancies in problem gambling treatment planning and public health strategies.

## Introduction

Understanding the psychological factors that contribute to problem gambling is essential to develop effective treatments, and public health strategies. One such factor that is consistently associated with problem gambling throughout recent research is emotion regulation (Alaba-Ekpo et al., 2024; Marchica et al., 2019; Velotti et al., 2021). Emotional dysregulation is a transdiagnostic process involving the awareness, understanding, and acceptance of experienced emotions, impulse control, goal directed behaviour, and the capacity to use strategies to modulate emotional responses (Gratz & Roemer, 2004). Having deficits in any of these areas is known as emotional dysregulation, which has been associated with the development and maintenance of gambling problems (Elmas et al., 2017; Jauregui et al., 2016; Velotti et al., 2021).

Several studies have shown that difficulties with emotion regulation is associated with problem gambling severity. For instance, Williams et al. (2012) examined emotional dysregulation in individuals receiving treatment for gambling disorder, a clinical comparison group comprised of individuals with mental health disorders (other than addictive disorders) and a healthy control group. The problem gambling group had greater difficulties with emotional awareness and emotion regulation strategies than both the clinical comparison and healthy control group. Additionally, problem gamblers had greater difficulties with emotional clarity and impulse control than the healthy control group. Jauregui et al. (2016) replicated these findings in a study comparing males with gambling disorder and healthy controls. Furthermore, this study found that problem gamblers had increased difficulties accepting emotions and engaging in goal-oriented behaviour than healthy controls. Similarly, differences in emotion regulation between problem gamblers and non-problem gamblers have also been found in community samples (Buen & Flack, 2022), young adults (Marchica et al., 2019; Orlowski et al., 2019), and high school students (Ciccarelli et al., 2021). Specifically, difficulties with emotion regulation have been found to be positively associated with problem gambling severity scores (Buen & Flack, 2022; Ciccarelli et al., 2021; Jauregui et al., 2016). Research by Orlowski et al. (2019) indicates that this may be in part due to problem gamblers experiencing greater difficulties tolerating negative emotions than non-problem gamblers, which is consistent with research on emotional avoidance and problem gambling (Riley, 2014). The recent increased focus on difficulties with emotion regulation in problem gambling has been captured in a meta-analysis by Neophytou et al. (2023), who found that 96% of studies included in their systematic review demonstrated a significant association between problem gambling and avoidant emotiol regulation strategies, suggesting that “problem gamblers use this behaviour to manage distress” (p. 137).

A noted limitation of the research literature surrounding emotion regulation and problem gambling is the role of gambling motives, with Neophytou et al. (2023) suggesting convergence of the gambling motives and emotion regulation literature to gain a more complete understanding of the mechanisms by which emotion regulation may influence problem gambling. Across the literature, proposed multidimensional models of gambling motivation have unveiled influential emotion-oriented reasons for gambling. For instance, The Gambling Motives Questionnaire (Stewart & Zack, 2008) includes gambling to escape negative emotions (coping motives), gambling to increase positive emotions (enhancement motives), and gambling for social rewards (social motives), with enhancement and coping motives predictive of problem gambling severity (Stewart & Zack, 2008). Gambling outcome expectancies (beliefs about what will happen when one gambles), are another way of capturing gambling for reasons related to experienced emotions, with escape and excitement outcome expectancies consistently positively associated with problem gambling severity (Flack & Morris, 2016; Flack & Buckby, 2020; Richardson et al., 2023). Recent research by Richardson et al. (2023) has shown escape expectancies appear more influential determinants of problem gambling in mixed-mode gamblers, whereas excitement expectancies are more so in offline-only problem gamblers, indicating that differing motives are important factors to consider when exploring problem gambling.

## Escape and Excitement

Gambling for escape is an avoidant coping strategy whereby individuals gamble to distract from negative emotional states (Riley, 2014). The role of escape in problem gambling is well established, as gambling to escape was previously a criterion of gambling disorder in the Diagnostic and Statistical Manual of Mental Disorders (DSM; 4th ed. text revision; American Psychiatric Association, 2000). Gambling for escape has been strongly linked to problem gambling across several studies (e.g., Marchica et al., 2020; Vaughan & Flack, 2022; Weatherly & Miller, 2013). For instance, Weatherly and Cookman (2014) found that over 32% of the variance in participant problem gambling scores was accounted for by escape motives. Similarly, coping motives (i.e., gambling to manage negative emotions) have been associated with problem gambling in community samples (Jauregui et al., 2016; Takamatsu et al., 2016) and clinical samples (Lambe et al., 2015). Additionally, laboratory studies have demonstrated that higher escape scores are associated with increased gambling engagement (Martner et al., 2012) and increased betting amount (Weatherly et al., 2010). These findings highlight that gambling to reduce negative emotions can increase the potential for gambling harm. Therefore, gambling for escape may be particularly risky for individuals with emotion regulation difficulties. However, few studies have directly examined the relationship between gambling for escape and emotional dysregulation. Weatherly and Miller (2013) found that gambling for escape was associated with deficits in emotion regulation in undergraduate students. Marchica et al. (2020) found that coping motives were associated with increased emotional dysregulation and problem gambling scores. These findings suggest that when individuals are unable to regulate their emotions, and gamble to escape these emotions, this places them at risk of problem gambling.

While gambling to escape negatively reinforces gambling behaviour, gambling to enhance emotional states positively reinforces gambling (Juodis & Stewart, 2016; Stewart & Zack, 2008). Enhancement motives or gambling for excitement have been found to predict gambling behaviours and problem gambling (Flack & Stevens, 2019; Obedzinski et al., 2019; Vaughan & Flack, 2022). Farhat et al. (2021) found that high school students who reported gambling for excitement had more permissive attitudes towards gambling and a higher risk of problem gambling. Similarly, Lambe et al. (2015) found that enhancement motives predicted gambling frequency, time and money spent gambling, and problem gambling scores in young adults. After controlling for gambling behaviour, enhancement motives remained the only significant predictor of problem gambling. Although there is a clear link between enhancement motives and problem gambling, the factors influencing this relationship have not been explained. One possibility is that individuals gamble for excitement and enhanced positive emotions to regulate their emotions, as was found in Marchica et al. (2020), where enhancement motives were associated with increased emotional dysregulation and problem gambling scores in young adults.

## Current Study

Studies have consistently demonstrated a positive relationship between difficulties with emotion regulation and problem gambling severity (e.g., Buen & Flack, 2022; Marchica et al., 2019; Orlowski et al., 2019). Although the mechanisms through which this relationship operates are largely unexplored, it is likely that individuals with emotion regulation difficulties are motivated to gamble to alter their emotional state (Flack & Morris, 2015; Stewart & Zack, 2008), which increases their problem gambling risk (Flack & Stevens, 2019; Marchica et al., 2020; Vaughan & Flack, 2022). The current study aims to extend previous research by exploring the relationship between difficulties with emotion regulation, excitement and escape outcome expectancies, and problem gambling severity. Specifically, this study hypothesises that escape and excitement outcome expectancies mediate the relationship between emotion regulation difficulties and problem gambling severity.

## Method

### Participants and Procedure

Participants who gambled at least once a month on one or more forms of gambling were recruited via Australian wide social media advertisements. Upon survey completion, participants were provided with the opportunity to enter a prize draw to win one of four $25 eGift cards. An a priori power analysis for mediation (Monte Carlo Power Schoemann et al., 2017) was conducted to determine the required sample size. Based on two parallel mediators, a desired power of 80%, and moderate (*r* = .4) correlations between all variables, a minimum of 154 participants were required. A total of 197 participants who gambled at least once a month completed the online survey. After removing participants who failed at least one attention check, the final sample comprised 187 adults (50.3% male, 48.7% female; *M*_*age*_ = 41.07 years, SD = 15.8 years). Ethics approval was obtained from the [name omitted for review].

The majority of participants identified as Caucasian (77%), followed by 7.5% Indigenous Australian, 5.3% Asian, and 10.2% grouped as ‘other’. Over two-thirds of the population were employed (46.5% full-time, 16% part-time, 7% casual), and almost half the sample had attained a university degree (36.4% undergraduate, 9.1% postgraduate). Most participants engaged in more than one gambling activity monthly, with the most popular monthly activity (electronic gambling machines (EGMs; 58.3%), sports betting (44.9%), and horse racing (42.8%).

### Measures

#### Problem Gambling

The Problem Gambling Severity Index (PGSI; Ferris & Wynne, 2001) is a 9-item scale comprised of statements related to problem gambling (e.g., “have you bet more than you could really afford to lose?”), where participants are asked to respond on a 4-point Likert scale from never (0) to almost always (3) in reference to the previous 12 months. The PGSI total score is commonly used as a continuous measure of problem gambling and has demonstrated good internal consistency in similar studies (Buen & Flack, 2022; Vaughan & Flack, 2022). According to the scoring conventions (Ferris & Wynne, 2001), 46% of the sample were classified as problem gamblers, 22.5% as moderate-risk gamblers, 16% as low-risk gamblers, and 15.5% as non-problem gamblers.

#### Emotional Dysregulation

The Difficulties in Emotion Regulation Scale (DERS) was used to measure emotional dysregulation. The DERS was developed to assess a range of clinically relevant transdiagnostic deficits in emotion regulation (Gratz & Roemer, 2004). The measure assesses the respondent’s beliefs about their ability to regulate their emotions, and the outcomes of their attempts to regulate emotions, rather than measuring the strategies themselves. Items (e.g., “*When I’m upset*,* I feel out of control*”, “*I have difficulty making sense out of my feelings*”) are endorsed on a 5-point scale from almost never (0) to almost always (5). The DERS total score is derived by obtained by summing the scores of all items, with higher scores indicating greater emotion regulation difficulties. The DERS total has demonstrated good internal consistency in previous studies (Burton et al., 2022; Marchica et al., 2019).

#### Gambling Outcome Expectancies

The Gambling Outcome Expectancies Scale (GOES; Flack & Morris, 2015) is an 18-item multidimensional measure of the expected outcomes of gambling that captures both emotional and monetary motivations. It includes five outcome expectancies: *escape*,* excitement*,* social*,* money*, and *ego*. Given the focus on the anticipated emotional outcomes, the current study used the escape (4 items) and excitement (3 items) subscales, as is consistent with previous research (Vaughan & Flack, 2022). The *escape* dimension reflects the anticipated cognitive and emotional relief anticipated from gambling (e.g., gambling to escape negative emotional states). In contrast, the *excitement* dimension evaluates how emotionally stimulating gambling is perceived to be (e.g., gambling to increase positive emotions). Respondents endorse how much they agree with belief-based statements (e.g., “*gambling is about enjoying intensive feelings*”; “*gambling is a way to forget everyday problems*”) on a 6-point Likert scale from strongly disagree (1) to strongly agree (6). *Escape* and *excitement* subscale scores are obtained by averaging the scores of subscale items, with higher scores indicating greater endorsement of escape or excitement as reasons for gambling. The factor structure of the GOES has been supported in subsequent research (Flack & Stevens, 2019) and the subscales have displayed good internal consistency (Flack & Morris, 2016; Vaughan & Flack, 2022).

## Results

### Data Preparation and Analytical Plan

Variables of interest were screened for outliers, skewness and kurtosis using SPSS 29. Skewness and kurtosis statistics for age, PGSI, DERS, and GOES were acceptable with absolute scores below 1 (Field, 2017). Examination of residual statistics revealed one case with a Mahalanobis Distance of 18.69, which was greater than the critical value χ2 = 18.47, df = 4, α = 0.01. However, inspection of Cook’s Distance indicated no concern for multivariate outliers, with the variance inflation factor (VIF) and tolerances indicating no issues with multicollinearity.

**Table 1:** Descriptive statistics and correlations between emotional dysregulation, gambling outcome expectancies, and problem gambling

Variable	1	2	3	4	5	M	SD
1. PGSI	0.95					8.22	7.37
2. DERS	0.54**	0.95				91.05	27.11
3. Escape	0.60**	0.38**	0.89			3.58	1.31
4. Excitement	0.47**	0.44**	0.67**	0.79		3.93	1.12
5. Age	.-0.35**	− 0.30**	− 0.11	− 0.21**	-	41.07	15.8
6. Gender	0.04	0.12	0.14	0.04	0.03	-	

Note. **p < .01. Cronbach’s α is presented along the principal diagonal

The relationships between emotion regulation difficulties, gambling outcome expectancies, and problem gambling were investigated with a series of correlations, as shown in Table 1. The significant, positive correlation between the predictor (DERS) and outcome variable (PGSI), significant positive correlations between the potential mediators (escape and excitement) and problem gambling scores, and significant positive correlations between the potential mediators and DERS scores, indicated mediation effects could be tested (Hayes, 2022). Gender was not correlated with any other variable, though given age was correlated with PGSI and DERS scores, and escape and excitement, age was included as a covariate in the mediation model.

The mediation model was tested using the SPSS PROCESS macro (version 4.2; Hayes, 2022). This procedure generates estimates for the direct and indirect estimates of multiple mediation models and produces bootstrapped confidence intervals and standard errors. For the current study, percentile-corrected bootstrap confidence intervals were based on 5,000 samples and considered significant if they did not include zero.

### Mediation Model

**Fig. 1:**
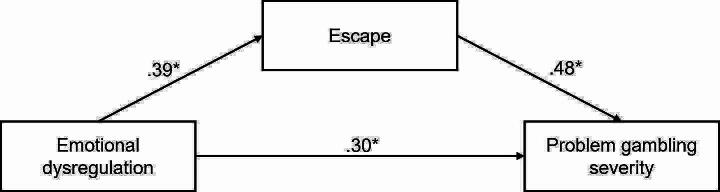
The multiple mediation model for emotional dysregulation and problem gambling severity. *Note*. *p <. 05. The covariate of age is omitted for clarity

The multiple mediation analysis indicated a significant indirect effect of emotional dysregulation on problem gambling through escape, though the indirect effect via excitement was not significant. Therefore, excitement was removed from the model and the analysis was conducted with escape as the single mediator. Figure 1 shows the standardised effects of the final mediation model.

Emotional dysregulation was found to partially influence problem gambling via escape. Although escape was a significant mediator, the direct relationship between emotional dysregulation and problem gambling severity remained significant. Therefore, the relationship between emotional dysregulation and problem gambling severity was partially mediated by escape. Table 2 shows the unstandardised direct, indirect, and total effects and the associated standard error and confidence intervals for the mediation model.

**Table 2:** Direct and indirect effects on problem gambling scores, controlling for age

Effect	Estimate	SE	95% CI	
			Lower	Upper
Indirect effect via escape	0.05	0.01	0.03	0.07
Direct effect (c’)	0.08	0.02	0.05	0.11
Total effect (c)	0.13	0.02	0.10	0.16

*R*^*2*^ = 0.33, *F*(2, 184) = 45.42, *p* < .001

## Discussion

Previous research has demonstrated that emotional dysregulation and the expected emotional benefits of gambling are associated with problem gambling. However, the influence of outcome expectancies on the relationship between emotion regulation difficulties and problem gambling has yet to be explored. The current study explored whether gambling outcome expectancies explained the relationship between emotional dysregulation and problem gambling in a community sample of regular gamblers. Specifically, the mediating effect of escape and excitement outcome expectancies on the relationship between emotional dysregulation and problem gambling was examined. The findings suggest that escape outcome expectancies explain the relationship between emotion regulation difficulties and problem gambling, though the mediating effect of excitement was not significant.

The positive association between emotional dysregulation and problem gambling found in this study is consistent with the current literature (Velotti et al., 2021). Additionally, emotional dysregulation was positively associated with escape and excitement outcome expectancies and problem gambling. These findings are consistent with previous research demonstrating that escape and excitement expectancies are associated with emotion regulation difficulties (Marchica et al., 2020; Weatherly & Miller, 2013) and problem gambling (e.g., Flack & Morris, 2015; Vaughan & Flack, 2022). The current study replicates the findings of Marchica et al. (2020) who demonstrated a positive relationship between emotional dysregulation, escape and excitement motives, and problem gambling. However, the current study differs from Marchica et al. (2020) as the anticipated consequences of gambling were measured in an adult sample of regular gamblers rather than the general motivations of gambling. Overall, the current study provides further evidence that emotion regulation difficulties and gambling to modulate emotional states are risk factors for problem gambling.

There was some support for the hypothesised mediation effect of escape and excitement on emotional dysregulation and problem gambling. That is, the outcome expectancy of escape partially mediated the relationship between emotional dysregulation and problem gambling. However, as this was only a partial mediation effect, emotional dysregulation does not influence problem gambling entirely through escape outcome expectancies. Although excitement was positively related to emotional dysregulation and problem gambling, it did not mediate this relationship. This finding indicates that when both excitement and escape are examined simultaneously, escape outcome expectancies exert the greater influence on problem gambling severity. This is consistent with Hagfors et al. (2023), who, in a longitudinal study of Finnish gamblers, found that both escape and excitement motives individually predicted problem gambling over time, with escape exhibiting the largest within-person effect. However, when all variables were included in their analyses, escape was the only significant predictor of problem gambling. Similarly, Flack and Stevens (2019) found that although both excitement and escape expectancies appear to become more influential at greater levels of problem gambling severity, only escape differentiated low-risk gamblers from problem gamblers.

Taken together, these findings suggest that escape expectancies exert a greater influence on problem gambling than excitement. The influence of escape on problem gambling may be more apparent with increased problem gambling severity. For instance, recent research by Mide et al. (2023) classified treatment-seeking problem gamblers by severity level, as outlined in the DSM (5th ed.; American Psychiatric Association, 2013). The study found that over 80% of severe problem gamblers reported gambling for escape, compared to 37.5% of moderate and 31% of mild problem gamblers. Additionally, moderate and severe problem gamblers had greater difficulties with emotion regulation than mild problem gamblers. These findings indicate that gambling to escape can be considered a consequence of emotion regulation difficulties, and both emotional dysregulation and gambling to escape emotional states should be considered as risk factors underlying problem gambling.

### Implications, Limitations, and Future Research

A strength of the current study is that the sample is relatively equal across genders (50.3% male, 48.7% female). This representation is important as research has shown higher rates of emotion regulation difficulties among women (Gardener et al., 2013), though men are often overrepresented in gambling studies (Marchica et al., 2019). In the current study, gender was not significantly correlated with any other variable, indicating that differences in emotion regulation and gambling risk across genders was not a confounding factor. An additional strength of the sample is the representation of problem gamblers. Compared to other cross-sectional research of regular gamblers (e.g., Barrault et al., 2017; Vaughan & Flack, 2022), the current sample had a relatively high percentage of problem gamblers (46%), However, the study sample is not representative of Australian gamblers at the population level (Australian Gambling Research Centre, 2023) due to differences in demographic characteristics and gambling behaviours. Therefore, caution should be exercised with the generalisability of these results to general community samples. Additionally, despite the sample comprising frequent gamblers, 54% of participants did not reach the cutoff scores for problem gambling. As such, sample characteristics differ from a clinical population, and the findings may not generalise to individuals with Gambling Disorder. Finally, given the cross-sectional design of the study, inferences of causality or directionality cannot be made. Specifically, it is unclear whether emotion regulation difficulties causes people to gamble for excitement and escape or if emotional dysregulation is a consequence of gambling to regulate moods. Future research employing longitudinal and experimental designs is required to investigate the nature of these relationships.

Another point of consideration given the present findings is the observation that problem gambling severity and gambling motivation vary across activities and modalities (Balodis et al., 2014; Barrault et al., 2019; Flack & Stevens, 2019; Goldstein et al., 2016). For instance, Barrault et al. (2019) found that individuals who participated in both strategic and non-strategic activities had higher problem gambling severity scores than those who only participated in strategic activities. Furthermore, activity type moderated the relationship between problem gambling and coping motives. Similarly, Richardson et al. (2023) found that in mixed-mode (i.e., both offline and online) gamblers, escape but not excitement outcome expectancies were predictive of problem gambling severity. In contrast, excitement but not escape was predictive of problem gambling for offline gamblers. Examining the role of gambling type on the relationship between emotional dysregulation, outcome expectancies, and problem gambling was beyond the scope of this study. However, given the important role of gambling type in the aetiology of problem gambling, future research is needed to investigate if these findings are consistent across gambling activities.

Despite limitations, the current study provides insight into the factors influencing problem gambling. The study findings have implications for the treatment of problem gambling. Given the role of emotional dysregulation in the development and maintenance of gambling problems, interventions targeting emotion regulation may be particularly beneficial for individuals who gamble to escape negative emotions. There is evidence that psychological therapies such as Dialectical Behaviour Therapy (Christensen et al., 2013; Korman et al., 2008) and those based on mindfulness techniques (e.g., (Maynard et al., 2018; McIntosh et al., 2016) reduce symptoms of gambling disorder by increasing emotion regulation and distress tolerance skills. The present findings also indicate problem gambling interventions may be more effective when the reasons for gambling are considered and may be specifically targeted. For instance, a review of innovative gambling disorder treatments by Snippe et al. (2019) describes a 6-session gambling intervention comprised of an augmented cognitive behavioural therapy approach (i.e., BEAT Gambling), that targeted maladaptive beliefs that were of relevance to participants, and showed promising preliminary results. Similarly, recent research (e.g., Dowling et al., 2022) has furthered development of personalised gambling interventions that incorporate ‘just-in-time’ adaptive behaviour change technique. It seems plausible that promoting generalised emotion regulation strategies with those that attempt to change more proximal, salient expectancies may confer synergistic effects in relation to achieving reductions in problem gambling.

## Conclusions

This study has contributed some convergence between the streams of research investigating emotional dysregulation, reasons for gambling (i.e., escape and excitement outcome expectancies), and problem gambling. Consistent with the current literature, higher levels of emotional dysregulation and escape and excitement expectancies were associated with higher problem gambling severity, with escape expectancies partially mediating the relationship between emotional dysregulation and problem gambling. Although excitement expectancies were hypothesised to have a mediating effect, they failed to explain the relationship between emotional dysregulation and problem gambling. The central finding that individuals with emotion regulation difficulties may gamble problematically to escape negative emotions makes an important contribution to the literature as it highlights the role of emotional dysregulation and outcome expectancies in the maintenance of problem gambling. Further research in this area is needed to enhance existing interventions and develop much needed tailored interventions for problem gamblers.

## Data Availability

The data supporting the findings in the current study are available from the corresponding author upon reasonable request.
